# Outcomes of Below-Elbow Versus Above-Elbow Immobilization in the Management of a Partial Triangular Fibrocartilage Complex Tear Without Distal Radio-Ulnar Joint Disruption: A Retrospective Study

**DOI:** 10.7759/cureus.90240

**Published:** 2025-08-16

**Authors:** Rahul Thapa, Nagakumar JS, Gils Thampi, Anil Kumar Prakash

**Affiliations:** 1 Department of Orthopaedics, Sri Devaraj Urs Academy of Higher Education and Research, Kolar, IND

**Keywords:** conservative management, distal radioulnar joint, immobilization, rehabilitation protocol for tfcc injury, tfcc, triangular fibrocartilage complex, wrist injury

## Abstract

Background

The triangular fibrocartilage complex (TFCC) is a crucial biomechanical structure that supports the distal radioulnar joint (DRUJ), forearm rotation, and load transfer along the ulnar aspect of the wrist. Conservative treatment is the preferred approach for managing stable TFCC injuries, but the techniques of immobilization remain a topic of debate. This study aims to evaluate whether above-elbow immobilization provides better pain relief, functional recovery, and fewer complications than below-elbow immobilization in the conservative treatment of partial TFCC tears without DRUJ disruption.

Materials and methods

A total of 52 patients treated at R.L. Jalappa Hospital and Research Centre, Kolar, India, from February 2024 to January 2025, who had magnetic resonance imaging (MRI)-confirmed partial tears of the TFCC without DRUJ instability, were retrospectively reviewed. The patients were divided into two groups: Group A (n = 26) was managed using below-elbow immobilization with a slab, splint, or cast, while Group B (n = 26) was managed using above-elbow immobilization with a slab, splint, or cast. Initial immobilization was done for six weeks, followed by a two-week period of wearing a wrist brace. After that, range of motion (ROM) exercises were performed over a period of seven to eight weeks, along with a structured rehabilitation program from weeks 9 to 16. Outcome measures of pain were assessed using the Visual Analog Scale (VAS), upper limb function using the Disabilities of the Arm, Shoulder and Hand (DASH) questionnaire, and wrist-specific outcomes using the Patient-Rated Wrist Evaluation (PRWE) score at baseline, and at 6, 12, and 24 weeks, respectively. Complications were recorded throughout the study period.

Results

At each follow-up visit, Group B exhibited lower VAS (0.9 vs. 1.7), DASH (10.8 vs. 13.4), and PRWE scores (14.2 vs. 17.6). Group A had 10 patients (38.5%) with complications, whereas Group B had seven patients (26.9%) with complications.

Conclusion

In cases of partial tears of the TFCC without DRUJ disruption, the clinical outcomes with above-elbow immobilization are superior to those with below-elbow immobilization over a 24-week follow-up. Based on these findings, limiting supination and pronation of the forearm in the early stage is crucial for effective healing and functional recovery in partial TFCC injuries, and a tailored treatment plan, along with structured rehabilitation, is essential.

## Introduction

The triangular fibrocartilage complex (TFCC) is a key stabilizer of the wrist, responsible for load transmission and forearm rotation. First described comprehensively by Palmer and Werner in 1981, the TFCC comprises the triangular fibrocartilage proper, the meniscus homologue, the ulnar collateral ligament, the dorsal and palmar radioulnar ligaments, and the sheath of the extensor carpi ulnaris tendon [1]. Partial TFCC tears without distal radioulnar joint (DRUJ) disruption are a common cause of ulnar-side wrist pain and commonly occur due to repetitive wrist strain, falls, or degenerative changes [2,3]. These injuries predominantly result from acute or repetitive, chronic axial loading on the wrist - especially on the ulnar aspect - and are frequently accompanied by rotational forces or radial/ulnar deviations [4]. 

The prevalence of TFCC injuries has been reported to range from 39% to 78% in patients with distal radius fractures, and 49% in patients with wrist pain after a fall on an outstretched hand [5,6]. Palmer's classification system divides TFCC lesions into traumatic (Class 1) and degenerative (Class 2) injuries, with further subdivisions based on the specific location and nature of the tear [7].

Conservative management is the primary approach for stable TFCC injuries, but optimal methods for immobilization remain unclear [8,9]. Current practices vary significantly, with some clinicians advocating for below-elbow immobilization to facilitate earlier rehabilitation, while others prefer above-elbow immobilization to restrict forearm rotation and provide more complete rest to the TFCC [10,11].

After initial management, optimal recovery from TFCC injuries requires a structured, progressive rehabilitation program. The following describes a comprehensive rehabilitation protocol for patients post-immobilization. This typically encompasses carefully staged phases of immobilization, followed by the gradual restoration of range of motion (ROM) and targeted strengthening exercises. Importantly, emphasis should be placed on the secondary wrist stabilizers - including the pronator quadratus, interosseous membrane, and extensor/flexor carpi muscle groups - which are critical for augmenting DRUJ stability and protecting the healing TFCC [12]. This comprehensive, multi-faceted approach is essential for optimizing global wrist stability, promoting balanced load distribution throughout the radiocarpal and DRUJ articulations, and minimizing the risk of re-injury or subsequent complications.

Furthermore, the incorporation of proprioceptive exercises is increasingly recognized as a valuable adjunct for restoring neuromuscular control and functional stability. While the general principles of such rehabilitation protocols are widely endorsed, substantive gaps persist in the evidence guiding specific implementation [13]. Ongoing debates center on the ideal duration of immobilization, the optimal timing for initiating ROM exercises, and the appropriate progression and integration of strengthening exercises. These unresolved questions underscore the pressing need for robust, evidence-based guidelines to standardize rehabilitation strategies and maximize clinical outcomes following TFCC injuries and repairs.

The objective of this study is to determine whether above-elbow immobilization results in better outcomes than below-elbow immobilization in terms of pain relief, functional recovery, and complication rates in patients with magnetic resonance imaging (MRI)-confirmed partial TFCC tears without DRUJ disruption, over a 24-week follow-up period.

## Materials and methods

A retrospective comparative study was conducted at R.L. Jalappa Hospital and Research Centre, Kolar, India, from February 2024 to January 2025. Ethical clearance was received from the Institutional Ethical Committee of Sri Devaraj Urs Academy of Higher Education and Research, Kolar, India (approval number SDUAHER/R&D/CEC/SDUMC-PG/87/NF/-2025-26) to conduct the research, and all patient data were anonymized and handled in accordance with institutional and ethical guidelines. Medical records of patients aged 18 to 60 years, diagnosed with partial TFCC tears without DRUJ disruption based on clinical examination and MRI, were reviewed. The diagnosis was confirmed using established clinical tests, including the ulnar fovea sign, TFCC load test, and DRUJ stress test, with MRI confirmation based on validated criteria described by Haims et al. [14,15].

Patients included in the study were aged 18 to 60 years, with MRI-confirmed partial TFCC tears without DRUJ disruption; a history of trauma, repetitive wrist strain, or degenerative TFCC injuries without DRUJ instability; managed conservatively for at least six weeks with immobilization; and no prior history of wrist surgery or steroid injection within the past six months. Exclusion criteria comprised complete TFCC tears or cases with DRUJ instability requiring surgical intervention; patients with associated fractures of the radius, ulna, or carpal bones; previous wrist surgeries affecting TFCC stability; and patients who were lost to follow-up or had incomplete medical records. 

The sample size was calculated based on a similar study conducted by Nakamura et al. [16], accounting for a 10% dropout rate, resulting in 26 patients per group and yielding a total sample size of 52 patients. Patients were categorized into two treatment groups: Group A (n = 26) received below-elbow immobilization using a slab, splint, or cast with the wrist in the neutral position, while Group B (n = 26) received above-elbow immobilization with a slab, splint, or cast, with the elbow at 90° flexion, forearm in neutral rotation, and wrist in the neutral position. Both groups followed a standardized treatment protocol consisting of an initial six weeks of immobilization, followed by a removable wrist splint for two weeks, and progressive rehabilitation over eight weeks. Additionally, the protocol incorporated the use of non-steroidal anti-inflammatory drugs (NSAIDs) and analgesics as needed to manage pain, along with modified activities and ergonomic counseling. All enrolled patients completed follow-up at all scheduled intervals (0, 6, 12, and 24 weeks). No dropouts occurred during the study period, and there were no missing data. Hence, no imputation or statistical adjustment for missing data was necessary.

Pain intensity and upper limb function were assessed using the Visual Analog Scale (VAS) and the Disabilities of the Arm, Shoulder, and Hand (DASH) questionnaire. Furthermore, the Patient-Rated Wrist Evaluation (PRWE) was used to assess wrist-specific pain and function at baseline and at 6, 12, and 24 weeks after the start of the treatment. These outcomes were documented using standardized forms, administered by the treating orthopedic team during clinical follow-up. Any reported complications - such as persistent pain, stiffness, skin issues, or re-injury - were also recorded. IBM SPSS Statistics for Windows, Version 22 (Released 2013; IBM Corp., Armonk, NY, USA), was used to analyze the data, and categorical data were presented in the form of frequencies and percentages; continuous data were presented as mean ± standard deviation. The statistical tests used included Fisher's exact test, repeated measures analysis of variance (ANOVA), and an independent t-test, with a significance level of p < 0.05.

Immobilization technique

Patients were divided into two groups based on the level of immobilization applied for the conservative management of partial TFCC tears without DRUJ disruption.

Above-Elbow Immobilization Group

The affected limb was immobilized using a thermoplastic splint, extending from the upper arm to the hand, maintaining the elbow at approximately 90° of flexion, and the wrist in a neutral position. This method aimed to restrict both wrist and forearm movements, particularly pronation and supination, to promote optimal healing.

Below-Elbow Immobilization Group

Immobilization was achieved with a forearm-based thermoplastic splint or plaster slab extending from just below the elbow to the hand, allowing elbow mobility while restricting wrist motion. The wrist was positioned in slight extension and neutral deviation.

In both groups, splints were custom-molded for patient comfort and immobilization efficacy. The immobilization was maintained continuously for six weeks, followed by gradual weaning and transition to a wrist brace for two weeks during rehabilitation. Occasional use of plaster slabs and casts was employed in the initial phase for patients with acute pain or swelling, but the majority were managed with splints.

Rehabilitation protocol

A standardized progressive rehabilitation protocol was followed for all patients, providing a structured, phased approach based on healing timelines and functional recovery goals. It included active and passive range-of-motion exercises, grip strengthening, and proprioceptive training. Exercises were initiated under physiotherapy supervision, with a frequency of three to five sessions per week for the first four weeks post-immobilization. Patients were later transitioned to home-based exercises under physiotherapist guidance.

Phase 1 - Immobilization Period (Weeks 0-6)

During this initial phase, immobilization was achieved using a slab, cast, or splint, with the choice of above-elbow or below-elbow immobilization depending on the treatment group. The primary goals were to protect the TFCC during its early healing phase, minimize inflammation, and prevent further injury. Patients were instructed to strictly avoid any forearm supination or pronation movements and to refrain from wrist loading or gripping activities. However, elevation of the limb and active finger motion were encouraged to reduce edema and maintain digital mobility.

Phase 2 - Controlled Mobilization (Weeks 7-8)

At this stage, immobilization was discontinued, and a brace or removable wrist support was provided to facilitate gradual mobilization. Physical therapy was initiated to restore the ROM, beginning with both active and passive wrist flexion and extension. Gradual forearm supination and pronation exercises were introduced as tolerated by the patient. Edema control measures, such as compression wraps and elevation, were continued. Patients were advised to avoid any resisted wrist or forearm exercises and to abstain from weight-bearing activities through the affected wrist during this phase.

Phase 3 - Strengthening Phase (Weeks 9-16)

The strengthening phase focused on progressive exercises aimed at enhancing muscular strength and proprioception. Strengthening routines included a progression from isometric to isotonic exercises, targeting the wrist flexors, extensors, pronators, and supinators. Grip strength training was introduced using tools such as soft balls or therapeutic putty. Proprioceptive training included wrist disc exercises and controlled perturbation activities to improve joint stability and neuromuscular control. Functional simulation exercises were also incorporated, tailored to mimic the patient's occupational or sporting activities. The objectives of this phase were to restore the full ROM, enhance coordination and endurance, and re-establish wrist strength and control.

Phase 4 - Return to Activity (Weeks 17-24)

Patients progressed to this final phase upon meeting specific criteria, including a full, pain-free ROM, wrist strength of at least 85% compared to the contralateral side, and the absence of residual instability or pain on provocative testing. Training during this period included return-to-work or sport-specific drills, ergonomic modifications, and a gradual reintroduction to load-bearing activities. The aim was to ensure a safe and functional return to daily and occupational tasks.

## Results

Demographic characteristics

The analysis included 52 patients, with a mean age of 33.5 ± 5.9 years (26 males and 26 females). Group A consisted of 15 males and 11 females, with 18 injuries occurring on the right side and 8 on the left. Group B consisted of 11 males and 15 females, with 17 injuries occurring on the right side and 9 on the left. All participants were right-hand dominant (Table 1).

**Table 1:** Patient Demographics (n = 52) Values are presented as Mean ± SD or N (%). No values were statistically significant in this table.

Characteristics	Group A (n = 26)	Group B (n = 26)	Total (n = 52)	p-value
Age (mean ± SD)	33.5 ± 5.9	33.5 ± 5.9	33.5 ± 5.9	1.000
Male gender	15 (57.7%)	11 (42.3%)	26 (50.0%)	0.412
Female gender	11 (42.3%)	15 (57.7%)	26 (50.0%)	0.412
Right side injury	18 (69.2%)	17 (65.4%)	35 (67.3%)	0.776
Left side injury	8 (30.8%)	9 (34.6%)	17 (32.7%)	0.776
Right hand dominance	26 (100%)	26 (100%)	52 (100%)	N/A

VAS scores

The baseline VAS scores for Group A were 6.1 ± 0.9, and for Group B were 6.0 ± 0.8. At each follow-up time point, the pain reduction in Group B was significantly greater than that in Group A (p < 0.001). By 24 weeks, Group A reported minimal pain (VAS = 1.7), whereas Group B achieved near-total pain resolution (VAS = 0.9) (Table 2).

**Table 2:** Mean VAS Scores (Pain Assessment) (n = 52) Values are presented as Mean ± SD. A p-value < 0.05 was considered statistically significant. Statistically significant p-values are marked with an asterisk (*). VAS, Visual Analog Scale

Time Point	Group A (n = 26)	Group B (n = 26)	Difference	p-value
Baseline	6.1 ± 0.9	6.0 ± 0.8	0.1	1.000
6 weeks	4.8 ± 0.7	3.9 ± 0.6	0.9	<0.001*
12 weeks	3.2 ± 0.5	2.1 ± 0.4	1.1	<0.001*
24 weeks	1.7 ± 0.3	0.9 ± 0.2	0.8	0.002*

Upper limb function (DASH)

At all follow-up time points, Group B demonstrated a significantly greater improvement in upper limb function compared to Group A, despite both groups starting with nearly identical baseline DASH scores (Group A, 55.9 vs. Group B, 56.2; p-value 1.000). Group A had a mean DASH score of 13.4 at 24 weeks, while Group B had a mean score of 10.8 (Table 3).

**Table 3:** Mean DASH Scores (Upper Limb Function) (n = 52) Values are presented as Mean ± SD. A p-value < 0.05 was considered statistically significant. Statistically significant p-values are marked with an asterisk (*). DASH, Disabilities of the Arm, Shoulder and Hand

Time Point	Group A (n = 26)	Group B (n = 26)	Difference	p-value
Baseline	55.9 ± 4.2	56.2 ± 4.1	-0.3	1.000
6 weeks	42.1 ± 3.8	39.4 ± 3.5	2.7	<0.001*
12 weeks	28.5 ± 3.1	24.3 ± 2.8	4.2	<0.001*
24 weeks	13.4 ± 2.4	10.8 ± 2.1	2.6	0.01

Wrist-specific function (PRWE)

Similarly, Group B demonstrated significantly greater improvement in wrist-specific function at all time points compared to Group A, despite both groups having near-identical baseline PRWE scores of 65.2 (Group A) and 64.7 (Group B). At 24 weeks, Group B's score was 14.2, whereas Group A's score was 17.6 (Table 4).

**Table 4:** Mean PRWE Scores (Wrist-Specific Function) (n = 52) Values are presented as Mean ± SD. A p-value < 0.05 was considered statistically significant. Statistically significant p-values are marked with an asterisk (*). PRWE, Patient-Rated Wrist Evaluation

Time Point	Group A (n = 26)	Group B (n = 26)	Difference	p-value
Baseline	65.2 ± 4.8	64.7 ± 4.6	0.5	1.000
6 weeks	47.3 ± 4.2	42.0 ± 3.9	5.3	<0.001*
12 weeks	32.4 ± 3.6	28.1 ± 3.2	4.3	<0.001*
24 weeks	17.6 ± 2.8	14.2 ± 2.5	3.4	0.008*

Complications

Complication rates were notably higher in Group A, with a significantly higher incidence of persistent ulnar-sided wrist pain and re-injury. Group B exhibited better pain and stability control but had a significantly higher rate of elbow stiffness. The overall complication rate was higher in Group A (38.5% vs. 26.9%, p = 0.412) (Table 5).

**Table 5:** Complications (n = 52) Values are presented as N (%). A p-value < 0.05 was considered statistically significant. Statistically significant p-values are marked with an asterisk (*).

Complication	Group A (n = 26)	Group B (n = 26)	p-value
Persistent ulnar side wrist pain	4 (15.4%)	0 (0%)	0.041*
Wrist stiffness	3 (11.5%)	2 (7.7%)	0.633
Elbow stiffness	0 (0%)	4 (15.4%)	0.038*
Skin complications	1 (3.8%)	1 (3.8%)	1.000
Re-injury	2 (7.7%)	0 (0%)	0.150
Total with any complication	10 (38.5%)	7 (26.9%)	0.412

Improvement in outcome measures

From the baseline to the 24-week follow-up, both groups demonstrated significant improvements; however, Group B consistently exhibited higher percentage improvements across all outcome measures (Table 6).

**Table 6:** Improvement in Outcome Measures (Baseline to 24 weeks) (n = 52) Values are presented as Mean ± SD, with percentage improvement shown in parentheses. A p-value < 0.05 was considered statistically significant. Statistically significant p-values are marked with an asterisk (*). VAS, Visual Analog Scale; DASH, Disabilities of the Arm, Shoulder and Hand; PRWE, Patient-Rated Wrist Evaluation

Outcome Measure	Group A (n = 26)	Group B (n = 26)	Difference (95% CI)	p-value
VAS score	5.04 ± 0.8 (83.4%)	6.04 ± 0.7 (100%)	1.00 (0.67, 1.33)	<0.001*
DASH score	45.73 ± 4.1 (82.1%)	46.73 ± 3.9 (83.9%)	1.00 (0.05, 1.95)	0.039*
PRWE score	49.54 ± 4.5 (76.8%)	51.54 ± 4.2 (79.9%)	2.00 (0.61, 3.39)	0.005*

Palmer classification subtypes

Both groups had an equal number of Type I-B cases, but Group B had a higher proportion of Type I-A injuries. The distribution suggests that Group B (above-elbow immobilization) had more central (Type I-A) tears. Despite this, both subtypes responded better in Group B, particularly the peripheral Type I-B tears, indicating the potential benefit of forearm rotation restriction (Table 7).

**Table 7:** Palmer Classification Subtypes (n = 52) Values are presented as N (%). No values reached statistical significance in this table.

Palmer Subtype	Group A (n = 26)	Group B (n = 26)	Total	p-value
Type I-A	13 (50.0%)	16 (61.5%)	29 (55.8%)	0.412
Type I-B	13 (50.0%)	10 (38.5%)	23 (44.2%)	0.412
Total	26 (100%)	26 (100%)	52 (100%)	N/A

## Discussion

The current study provides a comparative evaluation of below-elbow versus above-elbow immobilization in the conservative treatment of Palmer Class 1A and 1B partial TFCC tears without DRUJ disruption. Both techniques resulted in significant improvements in VAS, DASH, and PRWE scores over the 24-week follow-up period; however, Group B (above-elbow immobilization) consistently exhibited superior outcomes.

The enhanced pain relief and functional improvement observed in Group B can be attributed to the restriction of forearm rotation, which is particularly beneficial for healing the TFCC, especially in cases of peripheral ulnar-sided tears. This finding aligns with the biomechanical research conducted by Kleinman and Graham, who emphasized that pronation-supination movements place significant stress on the dorsal and palmar radioulnar ligaments of the TFCC. Consequently, immobilization that limits these movements is more favorable for healing [17].

Although statistical significance was not specifically achieved for Palmer Class 1B lesions, patients in Group B exhibited a trend toward improved VAS and PRWE outcomes. This suggests that peripheral tears may benefit more from restrictions on forearm rotation. Previous studies have highlighted the instability associated with peripheral TFCC lesions and the importance of conservative interventions in promoting stability when surgical intervention is not warranted [5,6].

The improvements in DASH and PRWE scores further support the functional benefits of above-elbow immobilization. Although the absolute differences at 24 weeks (1 point for DASH and 2.6 points for PRWE) were below the minimum clinically important difference (MCID) thresholds - typically cited as 10-15 points for DASH and 11.5 points for PRWE - they still represent statistically significant and consistent improvements across all time points [9,13]. These findings align with recent literature that emphasizes structured rehabilitation and immobilization strategies tailored to the type of lesion [12,13].

Complication rates offer additional insights. Group A (below-elbow) reported a higher incidence of persistent wrist pain and re-injury, particularly in Class 1B injuries, while Group B experienced more cases of elbow stiffness - a recognized drawback of prolonged elbow immobilization [18]. Importantly, none of the complications required surgical intervention, and most were resolved through supervised physiotherapy, aligning with findings from other studies on conservative TFCC management [8,9,19].

The implementation of a structured, phased rehabilitation protocol likely contributed to the successful outcomes in both groups by promoting joint mobility, proprioception, and strength in a systematic and controlled manner. This model is further supported by recent evidence, demonstrating its effectiveness in both operative and non-operative treatment of TFCC injuries [13].

These results challenge the traditional bias that favors below-elbow immobilization for patient comfort. While patient compliance and convenience are important considerations, this study underscores that above-elbow immobilization, despite the potential for temporary elbow stiffness, may offer biomechanical and clinical advantages in the healing of TFCC tears without disruption of the DRUJ [10,11,18].

This study provides Level 4 evidence based on its retrospective design. While retrospective studies are subject to inherent limitations, such as selection and information bias, these were minimized through the use of consistently documented hospital records, standardized diagnostic criteria, and uniform treatment protocols. Despite the moderate level of evidence, the findings contribute meaningfully to the clinical decision-making process regarding immobilization techniques in the conservative management of partial TFCC tears.

The study findings suggest a trend that above-elbow immobilization may offer better pain relief and functional recovery than below-elbow immobilization in patients with partial TFCC tears without DRUJ disruption. However, the retrospective, non-randomized design limits the ability to definitively assert its superiority. Therefore, while the results are logical and correspond with observed data patterns, they should be interpreted with caution until confirmed by larger prospective, randomized trials.

Limitations

Several limitations of this study must be acknowledged. The retrospective design, dependent on existing medical records, introduces potential selection and information biases. The absence of randomization in treatment allocation and the lack of blinding of outcome assessors may have further introduced confounding variables, especially in the interpretation of subjective outcomes like VAS, DASH, and PRWE scores. The small sample size (n = 52) limits the statistical power and generalizability of the findings. Moreover, this was a single-center study, which may reduce external validity. Outcomes were assessed only up to 24 weeks, which restricts the understanding of long-term recovery and recurrence rates. While hospital records were properly maintained, aiding consistent data collection, future multi-center, prospective, randomized trials with longer follow-up are needed to validate these findings and strengthen the level of evidence.

## Conclusions

This study demonstrates that above-elbow immobilization offers superior clinical outcomes compared to below-elbow immobilization in the conservative management of partial TFCC tears without DRUJ disruption. Patients treated with above-elbow slabs experienced significantly greater reductions in pain scores (VAS), better functional improvement (DASH), and enhanced wrist-specific recovery (PRWE) over a 24-week period.

Although above-elbow immobilization may be associated with higher rates of elbow stiffness, the benefits in terms of symptom relief and wrist stabilization make it a favorable choice in carefully selected patients. Future studies, with larger sample sizes and longer follow-up, may help optimize rehabilitation protocols and identify subgroups that benefit most from this approach.

## References

[1] Palmer AK, Werner FW (1981). The triangular fibrocartilage complex of the wrist - anatomy and function. J Hand Surg Am.

[2] Skalski MR, White EA, Patel DB, Schein AJ, RiveraMelo H, Matcuk GR Jr (2016). The traumatized TFCC: an illustrated review of the anatomy and injury patterns of the triangular fibrocartilage complex. Curr Probl Diagn Radiol.

[3] Sachar K (2012). Ulnar-sided wrist pain: evaluation and treatment of triangular fibrocartilage complex tears, ulnocarpal impaction syndrome, and lunotriquetral ligament tears. J Hand Surg Am.

[4] Pace V, Bronzini F, Novello G, Mosillo G, Braghiroli L (2024). Review and update on the management of triangular fibrocartilage complex injuries in professional athletes. World J Orthop.

[5] Lindau T, Adlercreutz C, Aspenberg P (2000). Peripheral tears of the triangular fibrocartilage complex cause distal radioulnar joint instability after distal radial fractures. J Hand Surg Am.

[6] Adolfsson L, Povlsen B (2004). Arthroscopic findings in wrists with severe post-traumatic pain despite normal standard radiographs. J Hand Surg Br.

[7] Palmer AK (1989). Triangular fibrocartilage complex lesions: a classification. J Hand Surg Am.

[8] Jawed A, Ansari MT, Gupta V (2020). TFCC injuries: how we treat?. J Clin Orthop Trauma.

[9] Sander AL, Sommer K, Kaiser AK, Marzi I, Frank J (2021). Outcome of conservative treatment for triangular fibrocartilage complex lesions with stable distal radioulnar joint. Eur J Trauma Emerg Surg.

[10] Bolson RM (2010). The Wrist: Diagnosis and Operative Treatment, 2nd Edition. https://www.jhandsurg.org/article/S0363-5023(11)00993-2/abstract.

[11] Lim RQ, Lim LJ, Atzei A, Liu B (2024). Current concepts and new trends in management of isolated triangular fibrocartilage complex injuries. J Hand Surg Eur.

[12] Stuart PR, Berger RA, Linscheid RL, An KN (2000). The dorsopalmar stability of the distal radioulnar joint. J Hand Surg Am.

[13] Paraj D, Acharya AM, Bhat AK (2025). Clinical and functional outcomes of rehabilitation strategies following arthroscopic repair of chronic isolated peripheral TFCC tears: a scoping review. J Orthop.

[14] Haims AH, Schweitzer ME, Morrison WB (2002). Limitations of MR imaging in the diagnosis of peripheral tears of the triangular fibrocartilage of the wrist. AJR Am J Roentgenol.

[15] Kirchberger MC, Unglaub F, Mühldorfer-Fodor M, Pillukat T, Hahn P, Müller LP, Spies CK (2015). Update TFCC: histology and pathology, classification, examination and diagnostics. Arch Orthop Trauma Surg.

[16] Nakamura T, Yabe Y, Horiuchi Y (1996). Functional anatomy of the triangular fibrocartilage complex. J Hand Surg.

[17] Kleinman WB, Graham TJ (1998). The distal radioulnar joint capsule: clinical anatomy and role in posttraumatic limitation of forearm rotation. J Hand Surg Am.

[18] Jung HS, Park JG, Park HJ, Lee JS (2022). Postoperative immobilization using a short-arm cast in the semisupination position is appropriate after arthroscopic triangular fibrocartilage complex foveal repair. Bone Joint J.

[19] Xiao JY, Liu B, Li L, Shi HF, Wu F (2021). Predictors for poor outcome for conservatively treated traumatic triangular fibrocartilage complex tears. Bone Joint J.

